# Facial expression recognition through muscle synergies and estimation of facial keypoint displacements through a skin-musculoskeletal model using facial sEMG signals

**DOI:** 10.3389/fbioe.2025.1490919

**Published:** 2025-02-12

**Authors:** Lun Shu, Victor R. Barradas, Zixuan Qin, Yasuharu Koike

**Affiliations:** ^1^ Department of Information and Communications Engineering, Institute of Science Tokyo, Yokohama, Japan; ^2^ Institute of Integrated Research, Institute of Science Tokyo, Yokohama, Japan

**Keywords:** facial expression recognition, sEMG, muscle synergy, musculoskeletal model, facial keypoints estimation

## Abstract

The development of facial expression recognition (FER) and facial expression generation (FEG) systems is essential to enhance human-robot interactions (HRI). The facial action coding system is widely used in FER and FEG tasks, as it offers a framework to relate the action of facial muscles and the resulting facial motions to the execution of facial expressions. However, most FER and FEG studies are based on measuring and analyzing facial motions, leaving the facial muscle component relatively unexplored. This study introduces a novel framework using surface electromyography (sEMG) signals from facial muscles to recognize facial expressions and estimate the displacement of facial keypoints during the execution of the expressions. For the facial expression recognition task, we studied the coordination patterns of seven muscles, expressed as three muscle synergies extracted through non-negative matrix factorization, during the execution of six basic facial expressions. Muscle synergies are groups of muscles that show coordinated patterns of activity, as measured by their sEMG signals, and are hypothesized to form the building blocks of human motor control. We then trained two classifiers for the facial expressions based on extracted features from the sEMG signals and the synergy activation coefficients of the extracted muscle synergies, respectively. The accuracy of both classifiers outperformed other systems that use sEMG to classify facial expressions, although the synergy-based classifier performed marginally worse than the sEMG-based one (classification accuracy: synergy-based 97.4%, sEMG-based 99.2%). However, the extracted muscle synergies revealed common coordination patterns between different facial expressions, allowing a low-dimensional quantitative visualization of the muscle control strategies involved in human facial expression generation. We also developed a skin-musculoskeletal model enhanced by linear regression (SMSM-LRM) to estimate the displacement of facial keypoints during the execution of a facial expression based on sEMG signals. Our proposed approach achieved a relatively high fidelity in estimating these displacements (NRMSE 0.067). We propose that the identified muscle synergies could be used in combination with the SMSM-LRM model to generate motor commands and trajectories for desired facial displacements, potentially enabling the generation of more natural facial expressions in social robotics and virtual reality.

## 1 Introduction

In human-human interactions, facial expressions are often more effective than verbal methods and body language in conveying affective information (Mehrabian and Ferris, 1967; Scherer and Wallbott, 1994), which is essential in social interactions (Blair, 2003; Ekman, 1984; Levenson, 1994; Darwin, 1872; Ekman and Friesen, 1969). The importance of facial expressions has also been shown in human-robot interactions (HRI) (Rawal and Stock-Homburg, 2022; Stock-Homburg, 2022; Yang et al., 2008; Bennett and Šabanović, 2014; Fu et al., 2023; Saunderson and Nejat, 2019), which are poised to become widespread in service industries (Gonzalez-Aguirre et al., 2021; Paluch et al., 2020), educational areas (Belpaeme et al., 2018; Takayuki Kanda et al., 2004), and healthcare domains (Kyrarini et al., 2021; Johanson et al., 2021). In such social settings, the use of facial expressions in robots can influence the users’ cognitive framing towards the robots, providing perceptions of intelligence, friendliness, and likeability (Johanson et al., 2020; Gonsior et al., 2011; Cameron et al., 2018). Expressive robots can also promote user engagement (Ghorbandaei Pour et al., 2018; Tapus et al., 2012) and enhance collaboration (Moshkina, 2021; Fu et al., 2023), improving performance in a given task (Reyes et al., 2019; Cohen et al., 2017). Therefore, the development of robots with the ability to recognize and generate rich facial expressions could facilitate the application of social robots in daily life.

Due to the visual nature of facial expressions, most facial expression recognition (FER) systems use computer vision to detect faces and determine the presence of facial expressions (Liu et al., 2014; Zhang et al., 2018; Yu et al., 2018; Boughida et al., 2022). These systems have achieved high accuracy (Yu et al., 2018; Boughida et al., 2022) in the recognition of predefined expressions, but suffer from robustness issues stemming from the sensitivity of vision systems to environmental variables such as illumination, occlusion and head pose (Zhang et al., 2018; Li and Deng, 2020). On the other hand, robots (Toan et al., 2022; Berns and Hirth, 2006; Faraj et al., 2021; Pumarola et al., 2020; Asheber et al., 2016) and animation software (Zhang et al., 2022a; Guo et al., 2019; Gonzalez-Franco et al., 2020) have been developed to generate facial expressions for HRI systems, although it is unclear what features of the generated expressions are important for successful HRI, and the goodness of the expressions has not been evaluated systematically (Faraj et al., 2021). Research on the recognition and generation of facial expressions in robotics has heavily relied on the facial action coding system (FACS), which is a framework that catalogs facial expressions as combinations of action units (AUs), which relate facial movements to the actions of individual muscles or groups of muscles (Hager et al., 2002). However, FACS only provides a qualitative relationship between facial motions and muscle activations. Therefore, *ad hoc* methods based on empirical measurements and calculations are required to define the precise temporal and spatial characteristics of facial points (Tang et al., 2023).

The measurement of surface electromyography (sEMG) of facial muscles offers an alternative to understand the temporal and spatial aspects of facial expressions in detail, as it provides information about the activation of muscles. Some studies have explored the use of sEMG signals for FER, although the resulting performance is not yet comparable to the performance of established computer vision methods due to limitations in the collection of facial sEMG (Lou et al., 2020; Hamedi et al., 2016; Cha et al., 2020; Kehri et al., 2019; Mithbavkar and Shah, 2021; Chen et al., 2015; Gruebler and Suzuki, 2010; Egger et al., 2019). Furthermore, given that facial expressions result from the coordinated action of different muscles (AUs as described by FACS), muscle synergy analysis offers tools to analyze these coordinated actions when measuring facial sEMG. A muscle synergy is a group of muscles that shows a pattern of coordinated activation during the execution of a motor task. Similar to the concept of AUs in the facial expression domain, muscle synergies are hypothesized to serve as the building blocks of motor behaviors (d’Avella et al., 2003). In practice, muscle synergies are identified through dimensionality reduction methods applied on the sEMG data, with non-negative matrix factorization (NMF) being favored for its interpretability of the identified synergies, as it organizes synergies into a spatial component containing the contribution of individual muscles, and a temporal component that dictates the non-negative activation coefficients of each synergy during the task (Rabbi et al., 2020; Lambert-Shirzad and Van der Loos, 2017). Surprisingly, there is little research using muscle synergies for FER related tasks (Perusquia-Hernandez et al., 2020; Delis et al., 2016; Root and Stephens, 2003; Chiovetto et al., 2018). Here, we propose using muscle synergy analysis for the FER task by extracting muscle synergies from sEMG and using features of the synergy activation coefficients to classify different facial expressions.

sEMG can also be applied in facial expression generation tasks, as granular spatial and temporal information about the action of facial muscles can inform the design of robotic systems capable of generating facial expressions. In particular, sEMG can be exploited to build musculoskeletal models (MSM) that estimate a physical output such as muscle force or joint torque based on sEMG measurements. Such models have been leveraged to build controllers for robotic upper and lower limbs (Zhang et al., 2022b; Bennett et al., 2022; Lloyd and Besier, 2003; Rajagopal et al., 2016; Qin et al., 2022; Mithbavkar and Shah, 2019). However, in the problem of the generation of facial expressions, the output of interest is the deformation of skin caused by muscle action. The field of computer graphics has excelled in modeling facial skin deformations to generate 3D models of facial expressions (Kim et al., 2020; Sifakis et al., 2005; Zhang et al., 2001; Lee et al., 1995; Kähler et al., 2001). However, these advanced CG models do not address the relationship between sEMG signals and facial deformations, which are necessary to inform the design of expressive robots. Other studies have used convolutional neural networks to predict the position of facial landmarks from sEMG signals to generate facial expressions in a virtual reality (VR) environment, but these methods treat the relationship between muscle activity and facial motions as a black box, forgoing the functional relationship between them (Wu et al., 2021). Here, we combine techniques developed by the robotics and the computer graphics fields by modeling both muscles (Shin et al., 2009; He et al., 2022) and skin (Zhang et al., 2001) as coupled non-linear springs, allowing us to estimate the displacement of facial points based on muscle activations. Additionally, we found that combining the skin-musculoskeletal model with a linear regression model enhanced the estimation performance when compared to the performance of both models in isolation.

The paper is organized as follows: in Section 2, we present the Materials and Methods for developing the FER systems and the facial keypoint displacement estimation system. Sections 2.1–2.4 describe the experimental protocol we used to collect the sEMG signals in a facial expression task. Section 2.5 provides details on the development of two FER systems based on individual muscle and muscle synergies, respectively. Section 2.6 describes the development of the skin-musculoskeletal model (SMSM) and the SMSM enhanced with a linear regression model (SMSM-LRM) for estimating the displacement of facial keypoints. Section 3 outlines the results of the proposed approaches in the FER and facial keypoint displacement estimation. In Section 4, we discuss the results of the muscle synergy analysis, FER analysis, and the estimation of displacement of facial points. Finally, Section 5 provides conclusions and prospects for future work.

## 2 Materials and Methods

**FIGURE 1:**
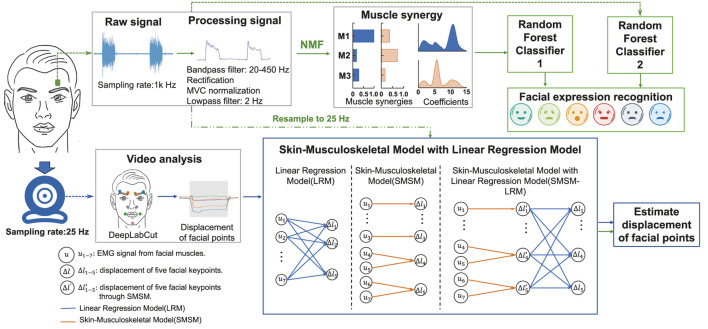
Methodology for recognition of facial expressions and estimation of displacement of facial points. Facial expression recognition: sEMG signals are measured through electrodes and bandpass filtered, rectified, normalized, and low-pass filtered. Subsequently, muscle synergies are extracted using non-negative matrix factorization (NMF), with the extracted synergy activation coefficients used for feature extraction. These features are then employed for facial expression recognition using a random forest classifier. Estimation of facial points displacement: displacements of facial points are measured using DeepLabCut, and these measurements, along with downsampled sEMG signals, are used to train the SMSM (Skin-Muscle-Skeletal Model), LRM (Linear Regression Model), and SMSM-LRM models.

We propose a methodology to use facial sEMG signals to recognize facial expressions and estimate the displacement of facial keypoints (Figure 1). We extracted muscle synergies from processed sEMG data of facial muscles, used their synergy activation coefficients for feature extraction, and used a random forest classifier for the classification of facial expressions. For comparison, we also built an RF classifier based on the same features, but extracted from processed sEMG signals. Then, we used the processed sEMG data and the displacement of facial keypoints measured from video data to develop and compare the performance of three models (SMSM, LRM, and SMSM-LRM) in estimating the displacements based on sEMG.

### 2.1 Participants

Ten participants (5 men), aged from 23 to 29 years old (mean age: 25.7 years (SD 2.7)), participated in the study after providing written informed consent. All the research procedures complied with the ethics committee of the Tokyo Institute of Technology and were conducted in accordance with the Declaration of Helsinki.

### 2.2 Experimental setup

Participants sat on a chair in front of a laptop computer and faced a webcam (resolution: 1280×720 , frame rate: 25 Hz). Participants were asked to face the webcam for the length of the experiment. We asked participants to sit upright and lean on the back of the chair during all experimental trials to keep a fixed distance from the webcam. During the experimental sessions, illustrations of target facial expressions were displayed on the laptop screen and participants were asked to replicate the target expression.

**FIGURE 2:**
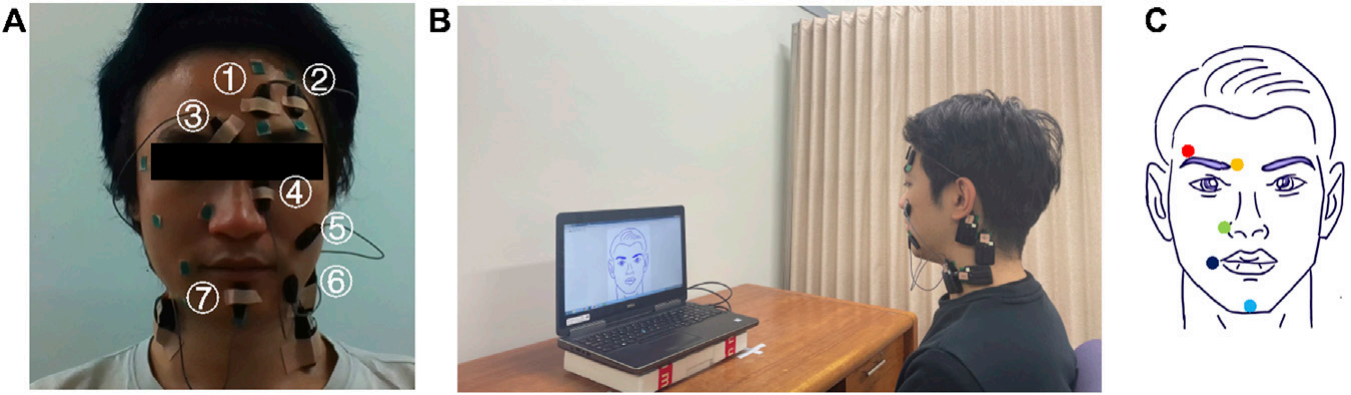
Experimental setup. **(A)** sEMG electrode placement. We used seven facial muscles: ① inner part of frontalis (inner frontalis, IF), ② outer part of frontalis (outer frontalis, OF), ③ corrugator supercilii (CS), ④ levator labii superioris alaeque nasi (LLSAN), ⑤ zygomaticus major (ZM), ⑥ depressor anguli oris (DAO), and ⑦ mentalis (Me). **(B)** Experimental setup. **(C)** Facial keypoints. We tracked the displacement of five facial keypoints during the experiment: outer eyebrow (red), inner eyebrow (orange), superior end of the nasolabial fold (green), mouth corner (dark blue), and chin (blue).

We recorded sEMG signals from seven facial muscle regions associated with the target facial expressions: inner frontalis region (IF), outer frontalis region (OF), corrugator supercilii region (CS), levator labii superioris alaeque nasi region (LLSAN), zygomaticus major region (ZM), depressor anguli oris region (DAO), and mentalis (Me). Hereafter, muscle names refer to their respective regions. Active bipolar electrodes were used to record EMG activity wirelessly (Trigno mini sensors, Trigno wireless system, Delsys) at a sampling rate of 1,024 Hz. Figure 2 shows the general placement of the electrodes. The placement process was meticulously standardized to ensure consistent and accurate signal collection across all participants. While healthcare professionals were not involved in this process, the accuracy of muscle identification and electrode placement was ensured through the use of established handbooks, anatomical atlases, and prior research literature. Additionally, the operator underwent extensive training under the guidance of experienced faculty members specializing in biotechnology and bio-interfaces, including theoretical sessions using established handbooks, anatomical atlases, and prior research literature. Initially, we identified the general areas, ranges, and actions of the targeted facial muscles (Cohn and Ekman, 2005; Mueller et al., 2022; Fridlund and Cacioppo, 1986; Hager et al., 2002; Kawai and Harajima, 2005). Especially, there is an atlas of EMG electrode placements for recording over major facial muscles (Fridlund and Cacioppo, 1986). Then, participants were asked to perform specific facial actions that engaged the targeted muscles while we manually palpated the expected location of each muscle, allowing us to pinpoint suitable sites for electrode placement. Before fixing the electrode placement, we temporarily placed electrodes at the identified sites and asked participants to perform the facial actions again. This step allowed us to monitor the EMG signals during each action to ensure that the muscle contraction produces measured EMG signals that match expectations. The positions were marked, compared with the atlas of EMG electrode placements (Fridlund and Cacioppo, 1986), and electrodes were then securely attached. This standardized procedure ensured that the data collected were both reliable and consistent. Because of electrode size and individual differences in participants’ facial structure, the electrodes were distributed differently for each participant. That is, in general, the electrodes were placed on the target muscle on different sides of the face, except for the muscle pairs IF and OF, and ZM and DAO, which were always placed on the same side of the face. The participants’ skin was cleaned before electrode placement to optimize the interface between electrodes and the skin.

The sEMG signal data was transferred to a laptop computer (Dell Precision 7510). Video of the participants’ facial expressions during the experiment was recorded using the laptop’s webcam to track the displacement of facial keypoints. We attached stickers on five facial keypoints (outer eyebrow, inner eyebrow, superior end of the nasolabial fold, mouth corner, chin) to use computer vision-based object-tracking software (DeepLabCut) to track their positions. Figure 2C shows the general position of the facial keypoints. These specific locations were standardized across all participants using a combination of the FACS (Hager et al., 2002), previous research (Mueller et al., 2022; Fridlund and Cacioppo, 1986; Kawai and Harajima, 2005) and established facial landmarks detection maps (Köstinger et al., 2011; Sagonas et al., 2016). The stickers were attached according to the electrode placement, such that the distribution of stickers across participants also varied (except for the outer and inner eyebrow points which were always attached to the same side of the face). The Lab Streaming Layer software (Stenner et al., 2023) was used to synchronize the sEMG and video data. The experimental routines were created using MATLAB (MathWorks, United States).

### 2.3 Experimental protocol

**FIGURE 3:**
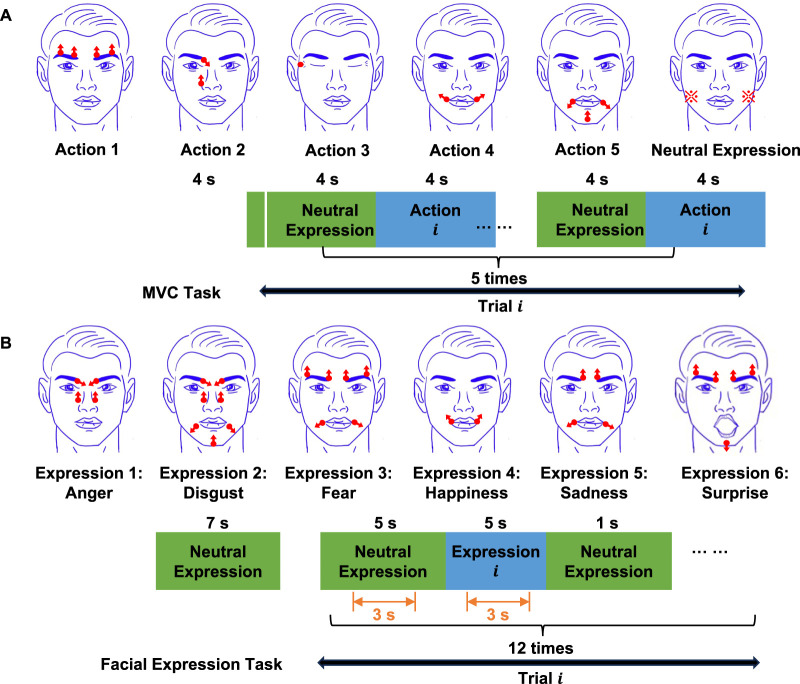
Structure of experimental tasks. **(A)** MVC task. i=1,2,3,4,5 , denotes the action index. In the ith trial, the participant started from the neutral state, performed the ith action, and returned to the neutral state. This was repeated five times. The screen displayed the target action to be performed. For the neutral state, participants were instructed to relax their masseter muscles, as indicated by the marks in the figure **(B)** Facial expression task. i=1,2,3,4,5,6 , denotes the six facial expressions separately: 1). anger, 2) disgust, 3) fear, 4) happiness, 5) sadness, and 6) surprise. In the ith trial, the participant started from the neutral expression, performed the ith target facial expression, and returned to the neutral expression. This was repeated 12 times. The screen displayed the target facial expression.

Before the main experiment, participants underwent comprehensive training to accurately perform six different facial expressions derived from the FACS system (anger, disgust, fear, happiness, sadness, surprise) as depicted in Figure 3B (Hager et al., 2002). These basic six facial expressions are universally common in different cultures (Ortony and Turner, 1990), although more recent research opposes this view (Jack et al., 2012). Nonetheless, these expressions have been extensively analyzed in both academic (Wolf, 2015; Küntzler et al., 2021) and applied settings (Rawal and Stock-Homburg, 2022; Tang et al., 2023) due to their role in human emotional communication. Therefore, our study uses this background to facilitate the comparison of results with past and future research. The training session consisted of the introduction stage and guided practice. In the introduction stage, we showed participants illustrations (Figure 3B) of each target facial expression, alongside verbal explanations on how to move different parts of the face to express the target facial expression. In the guided practice, participants were guided through each expression, receiving verbal cues to adjust their facial movements on how to correct the facial movement. During the training, we also monitored the EMG signals to ensure that only the muscles involved in the desired expression were activated. If we detected erroneous facial actions or EMG signals, we provided verbal cues to the participants to correct the action. It is a combination of subjective and more or less objective procedures. We evaluated that the expected Action Units (AUs) are moving, and that the expected EMG signals for the AUs are activated (without other AUs activating significantly).

In our study, the data acquisition and processing protocols were rigorously developed based on established methodologies within the field. To ensure the robustness of our procedures, we adhered closely to the protocols described in previous studies (Lou et al., 2020; Hamedi et al., 2016; Cha et al., 2020; Kehri et al., 2019; Chen et al., 2015; Mueller et al., 2022; Fridlund and Cacioppo, 1986). The main experiment consisted of two parts: the maximum voluntary contraction (MVC) task, and the facial expression task. In the MVC task, participants were asked to perform five different actions as intensely as possible to obtain MVC values for the recorded muscles. We used five actions to measure MVC: eyebrow elevation, eyebrow furrowing and nose elevation, eyelid closure, elevation of mouth corners, and depression of mouth corners (Cohn and Ekman, 2005). Figure 3A illustrates the structure of the MVC task. At the beginning of the task, the screen displayed a neutral expression, which participants maintained for 4 s. Then, the first trial started. A trial consisted of cycles of neutral expression and target actions. At the beginning of the first trial, the screen displayed the neutral expression for 4 s. Next, the screen displayed an illustration of the target action for 4 s. Participants were instructed to replicate the facial actions that were displayed on the screen at all times. This cycle was repeated 5 times within a single trial. The duration of a trial was 40 s. There were five trials in total. Each trial was associated to a different facial action.

In the facial expression task, participants were asked to perform six basic facial expressions included in the Facial Action Coding System: anger, disgust, fear, happiness, sadness, and surprise (Hager et al., 2002). Participants rested around 10 min between the MVC and facial expression task. Figure 3B illustrates the structure of the facial expression task. At the beginning of the task, the screen displayed the image of a neutral expression, which participants maintained for 7 s. Then the first trial started. A trial consisted of cycles of neutral and target facial expressions. First, the screen displayed the neutral expression for 5 s. Next, the screen displayed an illustration of the target expression for 5 s. Finally, the screen displayed the neutral expression again for 1 s. Participants were instructed to replicate the facial expression that was displayed on the screen at all times. This cycle was repeated 12 times within a single trial. The duration of a trial was 132 s. There were six trials in total. Each trial was associated to a different facial expression. In order to prevent muscle fatigue, participants were asked to rest 2 min between trials, and 10 min between two experiment tasks. After data collection, we reviewed the footage post-trial to identify and exclude any instances where the expressions were incorrectly performed.

### 2.4 Data processing

The sEMG data from both the MVC and facial expression tasks was filtered using a 20–450 Hz band-pass filter, rectified, and low-pass filtered using a 2 Hz cutoff frequency (Lou et al., 2020; Hamedi et al., 2016; Cha et al., 2020; Kehri et al., 2019; Mithbavkar and Shah, 2021; Chen et al., 2015; Gruebler and Suzuki, 2010; Egger et al., 2019). Additionally, before low-pass filtering, the sEMG data from the facial expression task was normalized using the maximum values of sEMG obtained in the MVC task.

We used the DeepLabCut (DLC) software (Mathis et al., 2018) to track five different facial keypoints: the inner eyebrow, outer eyebrow, nose, mouth corner, and jaw, to which we attached stickers (Figure 2C). First, we extracted 275 frames from a video sample (corresponding to 11 s) of each participant and manually labeled the stickers for training DLC. We also manually labeled one of the medial canthi and the upper point of both ears to use them as reference points, as they are immobile with respect to each other. This allowed us to compute a linear transformation that consistently aligned the extracted facial points in each frame to a canonical frame of reference (frontal view of the face), using the methods described in (Wu et al., 2021). Next, we used the trained DLC to track the facial keypoints in the rest of the video data and extracted the x and y coordinates of the facial keypoints. Finally, we calculated the displacement of the five facial keypoints during the task with respect to the canonical frame.

Because of the differing sampling rates between the sEMG signal (1 KHz) and the video data (25 Hz), we resampled the sEMG data to 25 Hz for the training of the model relating to sEMG and displacement of the facial keypoints. This resampling involved synchronizing the sEMG and video data using Lab Streaming Layer software. The experimental routines emitted trigger signals at the start and end of each expression, ensuring precise alignment. The sEMG signals were then downsampled by selecting one sample point every 40 ms based on these trigger points, reducing the sampling rate to match that of the video data. Additionally, there is noisy data caused by friction between the skin and the electrodes during the transitions between the neutral and target expressions. To eliminate this noise, we discarded 1 s of the data adjacent to the transitions. This applied to both the neutral and expression segments of both the sEMG and displacement data. Finally, to train the facial expression recognition system and the models for facial keypoint displacement estimation, we randomly selected one target expression from each trial. These periods were combined to create 12 reordered trials. Therefore, each reordered trial contained six different facial expressions in a randomized sequence.

### 2.5 Facial expression recognition system

We developed a facial expression recognition system that classifies participants’ expressions based on the synergy activation coefficients of muscle synergies extracted from the recorded facial muscles. This system relies on two procedures: muscle synergy extraction and facial expression classification.

#### 2.5.1 Muscle synergy extraction

We used the non-negative matrix factorization (NMF) method (Lee and Seung, 1999) to obtain muscle synergies and their synergy activation coefficients according to:

$$U=W_{s}\times C+E$$ (1)

where U is a m×t matrix ( m denotes the number of muscles, t denotes the number of samples) of recorded sEMG signals in all trials for each participant, $W_{s}$ is a m×s matrix of muscle synergies ( s denotes the number of synergies), C is a s×t matrix of synergy activation coefficients and E is a m×t matrix of unexplained variation in the muscle activations. In NMF, the number of muscle synergies s is a hyperparameter. We selected s so that the variance accounted for (VAF) by the extracted synergies $W_{s}$ reached at least 90%, which is a commonly used criteria in muscle synergy research (d’Avella et al., 2006; Turpin et al., 2021; Antuvan et al., 2016). We used the NMF implementation provided in the scikit-learn (Version: 1.3.2) package with the coordinate descent solver and Frobenius norm objective function, with multiple random initializations.

#### 2.5.2 Facial expression classification

We used a random forest classifier to classify facial expressions based on features derived from the muscle synergy activation coefficients C . We extracted features from isolated segments of the C synergy activation coefficients obtained by a sliding window of 150 ms (Nakayama et al., 2017) with a step of 40 ms. The 40 ms step was chosen to match the period defined by the 25 Hz video frame rate. The data contained in the sliding window was used to compute the classification features: root mean square (RMS), which measures the signal’s average power; variance (VAR), which measures signal fluctuation; mean absolute value (MAV), which measures the amplitude of the signal; and integrated EMG (IEMG), which measures increases in signal power and amplitude (Mithbavkar and Shah, 2019). The features in one sliding window are defined as Equations 2–5:

$$RMS_{j}=\sqrt{\frac{1}{R}\overset{R}{\underset{i=1}{\sum }}c_{i,j}^{2}}$$ (2)

$$VAR_{j}=\frac{1}{R}\overset{R}{\underset{i=1}{\sum }}{\left(c_{i,j}-\bar{c_{j}}\right)}^{2}$$ (3)

$$MAV_{j}=\frac{1}{R}\overset{R}{\underset{i=1}{\sum }}|c_{i,j}|$$ (4)

$$IEMG_{j}=\overset{R}{\underset{i=1}{\sum }}|c_{i,j}|$$ (5)

where $c_{i,j}$ represents the value of the synergy activation coefficient of muscle synergy j=1,2,3 (or sEMG signal of muscle j ) and R represents the number of sampled points in one sliding window (here, R=125 ). $\bar{c_{j}}$ represents the mean value of synergy activation coefficient of muscle synergy j=1,2,3 (or mean value of sEMG signal of muscle j ) in one sliding window. We also built a classifier that uses sEMG signals directly to classify the facial expressions using the same procedure as described above, with the values of the synergy activation coefficients replaced by the values of the EMG signals. Therefore, the input to the random forest classifier is an array of the features extracted from synergy activation coefficients or sEMG signal, that is, an array of s×4 elements for the classifier based on muscle synergies, and m×4 for the classifier based on sEMG signals.

The classifiers were trained based on pooled data from all participants without downsampling. The synergy activation coefficients or sEMG signals was separated into training and test sets. The training set contained a random permutation of 10 out of the 12 reordered trials per participant (see Section 2.4). We used five-fold cross-validation to train the classifiers. The remaining two reordered trials per participant were included in the test set. The training dataset waw in the shape of 100 trials, 3 synergies, 36,000 samples for the synergy-based classifier (100 trials, 7 muscles, 36,000 samples for the sEMG-based classifier), and the testing dataset was in the shape of 20 trials, 3 synergies, 36,000 samples for the synergy-based classifier (20 trials, 7 muscles, 36,000 samples for the sEMG signals-based classifier). Then we calculated the features extracted from sEMG signal and features from synergy activation coefficients to classify facial expressions. We used the *scikit-learn* package to implement the random forest classifiers with the parameters at their default values (setting the number of estimators to 100).

To evaluate classifier performance, we used the receiver operating characteristic (ROC) curve, F1-score, precision, recall, accuracy, and the confusion matrix. The ROC curve plots the true positive rate (TPR, also known as sensitivity or recall) against the false positive rate (FPR). The F1 score, defined as the harmonic mean of precision and recall, symmetrically incorporates the characteristics of both measures into one comprehensive metric. The confusion matrix visualizes algorithm performance, with rows indicating predicted classes and columns indicating actual classes.

### 2.6 Facial keypoint displacement estimation

We developed a skin-musculoskeletal model (SMSM) and a linear regression model (LRM), and combined them into a skin-musculoskeletal model with linear regression (SMSM-LRM) to estimate the displacement of facial keypoints using sEMG signals during the execution of facial expressions.

#### 2.6.1 Skin-musculoskeletal model

During the execution of a facial expression, facial muscles apply force on the skin, which produces skin deformation. Because skin is viscoelastic in its response to deformation (Zhang et al., 2001), it opposes the action of muscle force. Therefore, the forces generated by the contraction of facial muscles and those resulting from the deformation of skin are in a state of equilibrium. By integrating models of skin deformation with musculoskeletal models, we can delineate the relationship between muscle activation and skin deformation.

The relationship between stress and strain in the skin is non-linear, and can be modeled as a mass-spring-damper system with non-linear stiffness (Zhang et al., 2001). However, for simplicity, here we make three basic assumptions to model forces originating from skin deformation: 1. Skin stiffness is constant ( $K_{H}$ = 100 N/m), 2. The velocity of skin deformation is low, so that the dampening effect can be ignored, and 3. Skin deformation $\Delta l_{i}$ is equivalent to the displacement of an associated facial keypoint ( $\Delta l_{i}=x_{i}-x_{i0}$ , where $x_{i}$ is the current position of the facial keypoint i , and $x_{i0}$ is the neutral position of the facial keypoint i ). Following these assumptions, the force $f_{i}$ generated by the deformation of skin at point i , is:

$$f_{i}=K_{H}\Delta l_{i}$$ (6)

Next, to model forces produced by muscles, we use the Mykin model, which models muscles as springs with muscle activation-dependent stiffness and rest length (He et al., 2022; Shin et al., 2009). Thus, the force $F_{m}$ generated by muscle m is:

$$F_{m}=\left(k_{0}+k_{1}u\right)\left(l_{0}+l_{1}u-\Delta l_{m}\right)$$ (7)

where $k_{0}$ is the intrinsic stiffness of the muscle, $k_{1}$ is the muscle stiffness determined by muscle activation u , $l_{0}$ is the intrinsic rest length of the muscle, $l_{1}$ is a factor to determine the muscle rest length as a function of u , and $\Delta l_{m}$ is the current contraction length of the muscle.

To integrate the skin and musculoskeletal models, we classified the facial points measured experimentally (Section 2.2) as single-muscle systems or double-muscle systems (Hager et al., 2002; Waller et al., 2008). Single muscle systems included the outer eyebrow point with the outer frontalis muscle, the point on the superior end of the nasolabial fold with the levator labii superioris alaeque nasi muscle, and the point on the chin with the mentalis muscle. The outer frontalis elevates the outer eyebrow. The levator labii superioris alaeque nasi wrinkles the skin alongside the nose, elevating the position of the marker on the superior end of the nasolabial fold. The mentalis acts to depress and evert the base of the lower lip, while also wrinkling the skin of the chin, elevating the marker on the chin. On the other hand, the double muscle systems included the inner eyebrow point with the inner frontalis and corrugator supercilii muscle group, and the point on the corner of the mouth with the zygomaticus major and depressor anguli oris muscle group (Waller et al., 2008).

**FIGURE 4:**
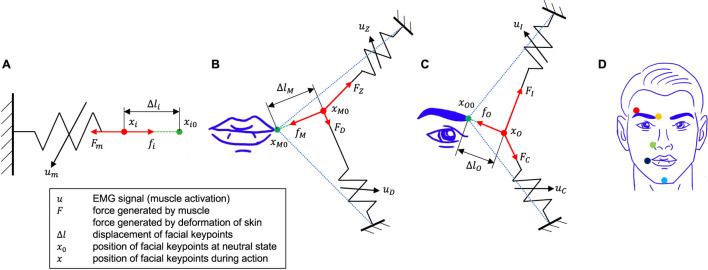
Skin-musculoskeletal models. **(A)** Single-muscle systems. A facial point is subject to $F_{m}$ , the force exerted by muscle m , and $f_{i}$ , the force exerted by skin deformation at facial point i . Muscle force is modeled as a spring with variable stiffness, as a function of muscle activation $u_{m}$ . Single muscle systems include the outer frontalis, levator labii superioris alaeque nasi, and mentalis. **(B)** Double-muscle system: skin-musculoskeletal model of the mouth corner. The mouth corner is subject to forces $F_{Z}$ and $F_{D}$ , generated by the zygomaticus major and depressor anguli oris muscles, respectively, and $f_{M}$ , the force generated by the skin deformation $\Delta l_{M}$ at the mouth corner. The neutral position of the mouth corner is given by $x_{M0}$ , and its current position by $x_{M}$ . **(C)** Double muscle system: skin-musculoskeletal model for the inner eyebrow. The inner eyebrow is subject to forces $F_{I}$ and $F_{C}$ , generated by the inner frontalis and corrugator supercilii muscles, respectively, and $f_{O}$ , the force generated by the skin deformation $\Delta l_{O}$ at the outer eyebrow. The neutral position of the outer eyebrow is given by $x_{O0}$ , and its current position by $x_{O}$ . **(D)** Facial keypoints, as shown in Figure 2.

For the single muscle systems, the structure of the skin-musculoskeletal model is illustrated in Figure 4A. In this case, the stretch length of the muscle, $\Delta l_{m}$ , and the deformation length of the skin, $\Delta l_{i}$ , are the same:

$$\Delta l_{m}=\Delta l_{i}$$ (8)

The neutral position for each single muscle system is defined as the position of the facial point when muscles are not activated. By combining Equations 6, 7, the force equilibrium equation is:

$$\left(k_{0}+k_{1}u\right)\left(l_{0}+l_{1}u-\Delta l_{m}\right)-K_{H}\Delta l_{i}=0$$ (9)

Therefore, according to the skin-musculoskeletal model, by combining the Equations 8, 9, the displacement of the facial point defined in each single muscle system (outer eyebrow, superior end of the nasolabial fold, and chin, as shown in Figure 4D) can be expressed as Equation 10:

$$\Delta l_{i}=\frac{\left(k_{0}+k_{1}u\right)\left(l_{0}+l_{1}u\right)}{K_{H}+\left(k_{0}+k_{1}u\right)}$$ (10)

In single-muscle systems $k_{0}$ , $k_{1}$ , $l_{0}$ , and $l_{1}$ are free parameters that are optimized to fit the experimental data as described in Section 2.6.4.

Figures 4B, C shows the structure of the skin-musculoskeletal model for double-muscle systems, that is, for the mouth corner muscle system, and the inner eyebrow muscle system. Here, we develop the skin-musculoskeletal model for the mouth corner system, but note that the resulting model is directly applicable to the inner eyebrow system. Even though the displacement of facial points in double-muscle systems is two-dimensional, here we assume that in the facial expression tasks, the direction of the displacement is highly biased in a single direction, allowing us to describe the displacement of the point as a one-dimensional quantity. Furthermore, the displacement of the facial point is associated with a change in the length of each of the two muscles in the system. For small displacement magnitudes, this relationship can be assumed to be linear. In the mouth corner system with the zygomaticus major and the depressor anguli oris muscles, this relationship can be expressed as:

$$\left\{\begin{array}{c} \Delta l_{Z}=\lambda_{Z}\Delta l_{M}\;\\ \Delta l_{D}=\lambda_{D}\Delta l_{M}\; \end{array}\right.$$ (11)

where $\Delta l_{Z}$ and $\Delta l_{D}$ are the changes in length of the zygomaticus major and depressor anguli oris muscles, respectively, $\Delta l_{M}$ is the displacement of the mouth corner, and $\lambda_{Z}$ and $\lambda_{D}$ are linear coefficients that depend on the geometry of the attachment of muscle and skin to the facial point. The muscle forces $F_{Z}$ and $F_{D}$ and the skin deformation force $f_{M}$ at the mouth corner are in equilibrium, as Equation 12:

$$F_{Z}+F_{D}+f_{M}=0$$ (12)

The magnitude of the force exerted by the skin can be expressed in terms of the magnitudes of the muscle forces as:

$$\left\|f_{M}\|=a_{Z}\|F_{Z}\|+a_{D}\|F_{D}\right\|$$ (13)

where $a_{Z}$ and $a_{D}$ denote projection coefficients of $F_{Z}$ and $F_{D}$ onto the direction of $f_{M}$ , which also depend on the geometry of the attachment of muscle and skin to the facial point, but for small displacements can be assumed to be constant. Inserting Equation 11 into Equations 6, 7, and the resulting expressions into Equation 13, the displacement $\Delta l_{M}$ of the corner of the mouth becomes Equation 14:

$$\Delta l_{M}=\frac{\sum_{i=Z,D}a_{i}\left(k_{0i}+k_{1i}u_{i}\right)\left(l_{0i}+l_{1i}u_{i}\right)}{K_{H}+\sum_{i=Z,D}a_{i}\lambda_{i}\left(k_{0i}+k_{1i}u_{i}\right)}$$ (14)

Following a similar procedure, the displacement of the inner eyebrow $\Delta l_{IE}$ can be expressed as Equation 15:

$$\Delta l_{IE}=\frac{\sum_{i=IF,CS}a_{i}\left(k_{0i}+k_{1i}u_{i}\right)\left(l_{0i}+l_{1i}u_{i}\right)}{K_{H}+\sum_{i=IF,CS}a_{i}\lambda_{i}\left(k_{0i}+k_{1i}u_{i}\right)}$$ (15)

In double-muscle systems $k_{0i}$ , $k_{1i}$ , $l_{0i}$ , $l_{1i}$ , $a_{i}$ , and $\lambda_{i}$ are free parameters that are optimized to fit the experimental data as described in Section 2.6.4.

#### 2.6.2 Linear regression model

We used a multivariate linear regression model (LRM) to relate the sEMG signals (or muscle activations) of all seven muscles to the displacements of all five facial points in the experiment. The LRM can be expressed as Equation 16:

$${\Delta L}_{\mathrm{LRM}}=W_{\mathrm{LRM}}U+\epsilon_{\mathrm{LRM}}$$ (16)

where $\Delta L_{\mathrm{LRM}}$ is a matrix of displacements of five facial points estimated by LRM (shape: [5,T] , T represents the number of samples), $W_{\mathrm{LRM}}$ is the weight matrix computed via linear regression (shape: [5,7] ), U is a matrix of the sEMG signal from all seven muscles in the experiment (shape: [7,T] ), and $\epsilon_{\mathrm{lrm}}$ is a matrix containing the residuals unaccounted by the linear regression. The weights in $W_{\mathrm{LRM}}$ are free parameters optimized as described in Section 2.6.4.

#### 2.6.3 Skin-musculoskeletal model with linear regression model

The SMSM does not take into account the effects of the displacement of facial points outside the single or double muscle systems to estimate the displacement of a given facial point. However, facial points may be connected to other facial points through skin, and thus may be subject to forces other than those considered in the single and double muscle systems. Here, we addressed this issue by combining the SMSM and the LRM to integrate their estimation capabilities. The skin-musculoskeletal model with linear regression (SMSM-LRM) can be expressed as Equation 17:

$$\Delta L=W\Delta l_{\mathrm{SMSM}}+\epsilon$$ (17)

where ΔL is the matrix of displacements of facial points estimated by the SMSM-LRM (shape: [5,T] ), W is a weight matrix computed by linear regression (shape: [5,5]), $\Delta l_{\mathrm{SMSM}}$ is a matrix of facial point displacements produced by the SMSM (shape: [5,T] ) and ϵ represents residuals unaccounted by the SMSM-LRM (shape: [5,T] ). The free parameters in the SMSM component of the model and the weights in W are optimized to fit the experimental data as described in Section 2.6.4.

#### 2.6.4 Training the SMSM, LRM and SMSM-LRM models

The free parameters in the SMSM, LRM, and SMSM-LRM were determined through iterative optimization within a supervised learning framework. We initialized a set of parameters which were subsequently refined across 18,000 epochs using gradient descent, facilitated by the Adam optimizer. The optimization process aimed to minimize the mean squared error between the estimations of the models and the actual data. The training data and test data sets were the same sets as those defined for the facial expression recognition task with downsampling. The training dataset was in the shape of 10 trials, seven muscles, 900 samples, and the testing dataset was in the shape of two trials, seven muscles, 900 samples per participant. We used 5-fold cross-validation to enhance the model’s generalizability and prevent overfitting. The model with the best performance across the five folds was selected for use on the test dataset.

#### 2.6.5 Evaluation methods

We evaluated the model’s performance using two standard metrics: the coefficient of determination $\left(R^{2}\right)$ and the normalized root-mean-square error (NRMSE) with respect to the difference in maximum and minimum values in the data. We assessed differences in the metrics associated with each model using ANOVA tests. We used Tukey’s Honestly Significant Difference (HSD) *post hoc* test to identify specific group disparities. We used the *stats* module from the *scipy* package to perform the statistical analysis.

## 3 Results

### 3.1 Muscle synergies allow low-dimensional visualization of facial muscle control

**FIGURE 5:**
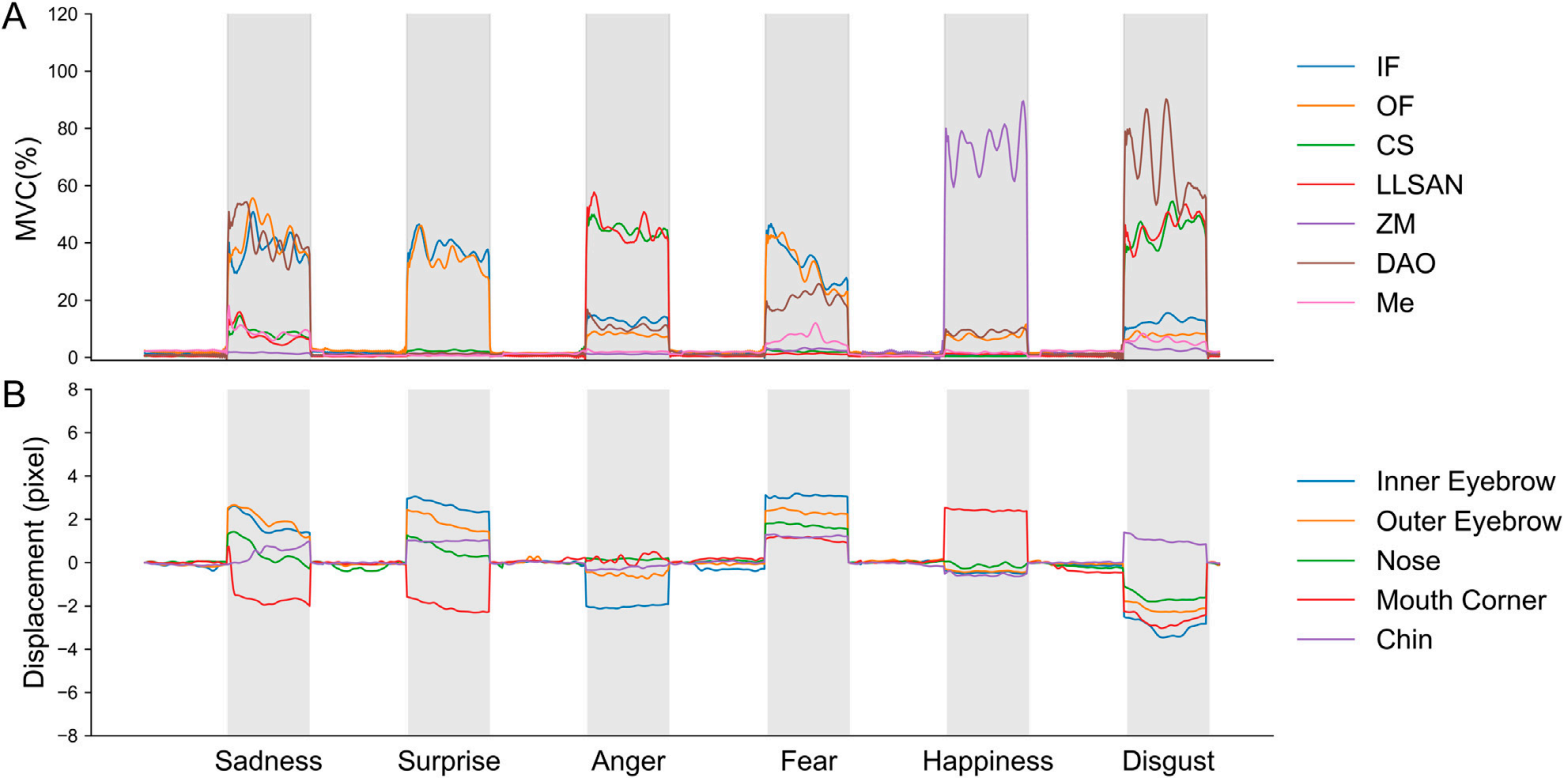
Normalized sEMG signals and displacement of facial keypoints during the facial expression task for a representative participant (participant 2). **(A)** Normalized sEMG signals of seven muscles during one reordered trial containing a single repetition of each facial expression. **(B)** Displacement of five facial key points during one reordered trial containing a single repetition of each facial expression.

**FIGURE 6:**
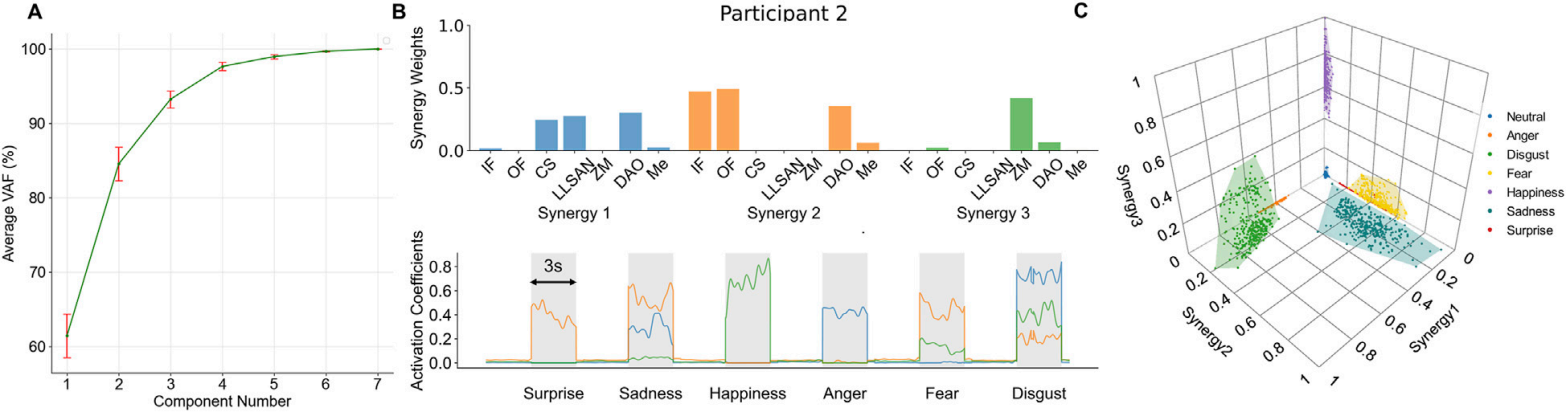
Muscle synergies of facial muscles during facial expressions. **(A)** VAF in the measured sEMG signals as a function of the number of synergy components extracted across all participants. Three synergies were enough to account for at least 90% of the variance in all participants. **(B)** Extracted synergy components for a representative participant (participant 2), and synergy activation coefficients for each synergy during a representative trial in the facial expression task (participant 2, trial 2). **(C)** Clusters of synergy activation coefficients in the 3-dimensional synergy space across all trials (participant 2). The shaded regions in the figure show the convex hulls containing the muscle synergy activations produced during each facial expression. The convex hulls are computed in three-dimensional space from the points representing the synergy activations and reflect the range and shape of each expression within the muscle synergy activation space. The convex hulls were calculated using the *ConvexHull* function from the *scipy* library.

The normalized sEMG signals and the displacement of the five measured facial keypoints in a reordered trial of the facial expression task of a representative participant are illustrated in Figure 5. These reordered signals were employed to extract both muscle synergies and relevant classification features for the classification of facial expressions. To determine the optimal number of muscle synergy modules, we computed the variance accounted for (VAF) with the synergy module number ranging from 1 to 7 per participant. For all participants, 3 synergies were enough to account for 90% of the variability in the muscle activation data (Figure 6A).

Figure 6B shows the muscle synergies and synergy activation coefficients for a representative participant in one of the reordered trials of the facial expression task. Synergy 1 primarily activated corrugator supercilii, levator labii superioris alaeque nasi, and depressor anguli oris; synergy 2 involved significant activation in inner and outer frontalis, depressor anguli oris, and mentalis; synergy 3 predominantly activated zygomaticus major and depressor anguli oris. We compared the extracted muscle synergies across all participants using the cosine similarity metric (Rimini et al., 2017; d’Avella and Bizzi, 2005). We found that the three extracted synergies were similar across participants, especially synergy 2 (average cosine similarity; synergy 1: 0.75 (SD 0.16), synergy 2: 0.87 (SD 0.10), and synergy 3: 0.72 (SD 0.20). Synergy 1 was predominantly activated during anger, disgust, and sadness expressions; synergy 2 was predominantly activated during fear, sadness, surprise and disgust expressions; synergy 3 was predominantly activated during disgust and happiness expressions. Figure 6C shows clusters of synergy activation coefficients in the three-dimensional synergy space for a representative participant. Interestingly, the synergy activation coefficients for the expressions of anger, surprise, and happiness are predominantly clustered around a single dimension of the synergy space for all participants. Anger is mainly associated with synergy 1, surprise with synergy 2, and happiness with synergy 3. The remaining expressions are associated mainly with combinations of two or three dimensions in the synergy space. For example, for participant 2, the expressions of disgust, sadness, and fear are clustered in regions of the synergy space spanning a combination of synergies 1, 2, and 3, synergies 1 and 2, and synergies 2 and 3, respectively. Results for the rest of the participants are provided in the Supplementary Material (Supplementary Figure S1).

### 3.2 Performance of synergy-based classification of facial expressions is good enough compared to sEMG-based classification

**FIGURE 7:**
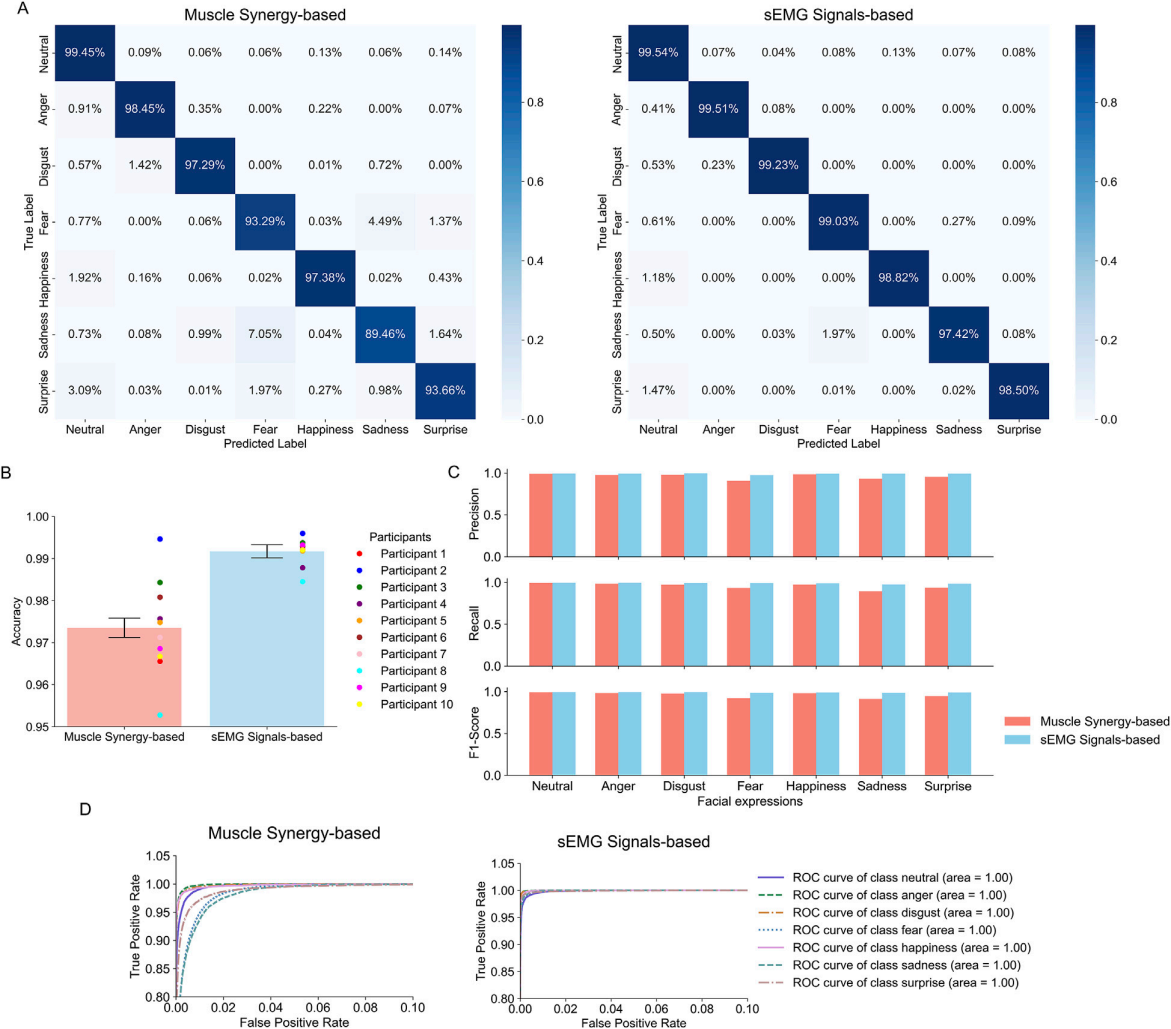
Evaluation metrics for the facial expression recognition systems. **(A)** Confusion matrices across all participants of the synergy- and sEMG-based classifiers. Each row and column represents the true labels and predicted labels, respectively. The intensity of the shade in each box is proportional to the displayed accuracy. **(B)** Average classification accuracy across all facial expressions and participants for the synergy- and sEMG-based classifiers. Error bars indicate the standard deviation in overall accuracy across all participants. Points represent the accuracy for individual participants. **(C)** Recall, precision, and F1 score of each facial expression across all participants for the synergy- and sEMG-based classifiers. **(D)** ROC curves for each expression for the synergy- and sEMG-based classifiers. The line representing the performance of a classifier with an Area Under the Curve (AUC) of 0.5 is not shown due to the scale of the axes.

Figures 7A–D shows the results of the facial expression classifier based on synergy activation coefficients and the classifier based on sEMG signals. The synergy-based classifier achieved expression-specific accuracies across all participants of 99.5% for the neutral expression (vs. sEMG-based classifier: 99.5%), 98.5% for anger (vs. 99.5%), 97.3% for disgust (vs. 99.2%), 93.3% for fear (vs. 99.0%), 97.4% for happiness (vs. 98.82%), 89.5% for sadness (vs. 97.4%), and 93.2% for surprise (vs. 98.5%), as shown in the confusion matrix (Figure 7A). Furthermore, the synergy-based classifier achieved average accuracies across all participants of 97.4% (vs. sEMG-based classifier: 99.2%) and accuracy for each participant of 96.6%, 99.5%, 98.4%, 97.6%, 97.5%, 98.1%, 97.1%, 95.3%, 96.7% and 97.5% (Figure 7B). Additionally, both classifiers maintain a high level of performance across all expressions, with most precision, recall, and F1 scores exceeding 0.9 (Figure 7C). Finally, the proximity of the ROC curve of each expression to the upper left corner of the graph indicates a high true positive rate (TPR) and a low false positive rate (FPR) (Figure 7D). Notably, all expressions exhibit an area under the curve (AUC) value close to 1 for both classifiers.

### 3.3 SMSM-LRM has the best performance in estimation of displacement of facial points

**FIGURE 8:**
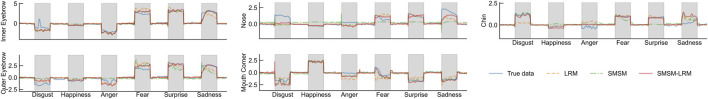
Estimation results of facial keypoints by SMSM, LRM, and SMSM-LRM (participant 2): the displacement of the inner eyebrow, the outer eyebrow, the nose, the mouth corner, and the chin, respectively. The blue lines represented the measured displacements of five facial points calculated based on DeepLabCut. The orange dashed, green dotted, and red lines represent the prediction results from the LRM, SMSM, and SMSM-LRM, respectively.

**FIGURE 9:**
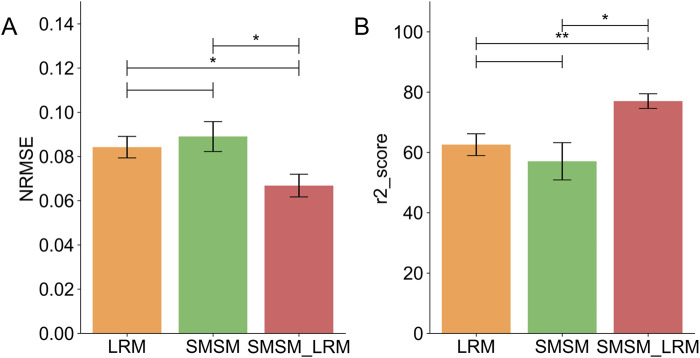
Comparisons of five-fold cross-validation results of all participants: **(A)** the average NRMSE of each model. **(B)** The average $R^{2}$ of each model.

Figure 8 presents representative results on the test set (participant 2) of the SMSM, LRM and SMSM-LRM models in the estimation of the displacements of five facial points defined in the experiment. We evaluated the performance of the three models by computing the coefficient of determination $\left(R^{2}\right)$ and Normalized Root Mean Square Error (NRMSE) (Figure 9) across participants. Notably, the SMSM-LRM model demonstrated the highest performance in predicting the displacements of all five facial points across all participants, according to both $R^{2}$ (77.02 (SD 2.45)) and NRMSE (0.0668 (SD 0.0051)), compared to the SMSM ( $R^{2}$ : 57.07 (SD 6.17), NRMSE: 0.0890 (SD 0.0068)) and LRM ( $R^{2}$ : 62.58 (SD 3.61), NRMSE: 0.0842 (SD 0.0049)) models. A one-way ANOVA revealed that there was a statistically significant difference in $R^{2}$ scores between at least two models (F (2, 27) = 5.5699, p = 0.0094). Tukey’s HSD Test for multiple comparisons found that the mean value of $R^{2}$ scores was significantly different between LRM and SMSM-LRM (p = 0.0242) and between SMSM and SMSM-LRM (p = 0.0182). However, there was no statistically significant difference between LRM and SMSM (p = 0.5715). Similarly, a one-way ANOVA revealed that there was a statistically significant difference in NRMSE between at least two models (F (2, 27) = 4.2775, p = 0.0243). Tukey’s HSD Test for multiple comparisons found that the mean value of NRMSE was significantly different between LRM and SMSM-LRM (p = 0.0243) and between SMSM-LRM and SMSM (p = 0.0120). However, there was no statistically significant difference between LRM and SMSM (p = 0.4542).

## 4 Discussion

In this study we defined two aims: 1. To establish a framework for recognizing facial expressions based on muscle synergies of facial muscles, and 2. To estimate the displacement of facial keypoints from the sEMG signals of the facial muscles as a step towards generating facial expressions in robotic systems. For the facial expression recognition task, we employed non-negative matrix factorization (NMF) to identify muscle synergies and their synergy activation coefficients from the measured sEMG of seven facial muscles, and used the synergy activation coefficients to train a random forest classifier to recognize six different facial expressions. For the facial expression generation task, we introduced the skin-musculoskeletal model combined with linear regression (SMSM-LRM) as a novel approach to estimate the displacements of five facial keypoints: inner eyebrow, outer eyebrow, superior end of the nasolabial fold, mouth corner, and chin, which we measured using video-based object tracking software. The FER system based on muscle synergies had a high accuracy in classifying facial expressions compared to existing methods, but a slightly lower accuracy than using an sEMG-based classifier. We also found that the proposed SMSM-LRM outperforms the SMSM and LRM in estimating the displacement of the facial keypoints.

### 4.1 Muscle synergy-based facial recognition system has good performance

Our facial expression recognition system based on muscle synergies yielded enhanced performance for all six facial expressions compared to previous research (Chen et al., 2015; Cha et al., 2020; Kehri et al., 2019; Mithbavkar and Shah, 2021; Gruebler and Suzuki, 2010). Notably, the recognition rates for the expressions of fear (93.3% vs. 65.4% (Cha and Im, 2022)), sadness (89.5% vs. 78.8% (Cha and Im, 2022)), surprise (93.7% vs. 88.9%) (Chen et al., 2015) and anger (98.5% vs. 91.7% (Chen et al., 2015)) observed considerable improvements. The main reason for this superior performance is likely that previous studies primarily focused on sEMG signals collected around the eyes, whereas our method expanded its scope to include sEMG signals collected around the mouth. For instance, the depressor anguli oris influences the motion of the mouth corner, which is useful to discern expressions of fear and sadness, enhancing the recognition accuracy of our system.

However, we found that a classifier based on sEMG from individual muscles outperforms the classifier based on muscle synergies (average accuracy: sEMG-based - 99.2% vs. muscle synergy-based - 97.4%). This aligns with the finding that the residuals E in the sEMG signals U that the identified muscle synergies $W_{s}$ cannot account for may contain task-relevant information (Equation 1) (Barradas et al., 2020). However, the difference in performance of the FER systems based on individual muscles and muscle synergies is small, and the performance of the muscle synergy-based system is considerably better than previously developed systems. Therefore, the muscle synergy-based FER system is good enough for this classification task.

Figure 6C and Supplementary Figure S1 show the contribution of each identified muscle synergy to the execution of each facial expression. Across all participants, the neutral expression is associated to a null activation of all three synergies, as expected. Interestingly, across all participants, synergies 1, 2, and 3 are each predominantly related to only one facial expression: anger, surprise, and happiness, respectively. On the other hand, the facial expressions of disgust, fear, and sadness are associated with combinations of synergies 1, 2, and 3. As shown in Figure 6C, participant 2 showed clearly separate clusters of synergy activation coefficients for each facial expression. However, for other participants, the clusters of expressions of fear, happiness, sadness, and surprise showed some overlap (Supplementary Figure S1). This is especially true for participants 1, 8, 9 and 10, resulting in lower recognition accuracy than for other participants (Figure 7B). We also found instances of global misclassification of facial expressions due to common synergies involved in the execution of different expressions. Particularly, the accuracy for fear, sadness, and surprise expressions did not exceed 95%. This may be because the activation of synergy 2 for these emotions is similar, given that synergy 2 predominantly activates the inner and outer frontalis muscle, which belongs to action unit (AU) 1 in the facial action coding system (FACS), and AU1 is known to be involved in these facial expressions (fear, sadness, surprise) (Tables 1, 2 (Hager et al., 2002).

**TABLE 1:** Facial expressions and corresponding action units (AU) (Hager et al., 2002).

Facial expression	Action units (AU)
Happiness	6 + 12
Sadness	1 + 4 + 15
Surprise	1 + 2 + 5 + 26
Fear	1 + 2 + 4 + 5 + 7 + 20 + 26
Anger	4 + 5 + 7 + 23
Disgust	9 + 15 + 16

**TABLE 2:** Action units (AU) and corresponding muscles (Hager et al., 2002).

AU number	Description	Muscle
1	Inner Brow raiser	Frontalis* (pars medialis)
2	Outer brow raiser	Frontalis* (pars lateralis)
4	Brow lowerer	Depressor Glabellae, Depressor Supercilli, Corrugator Supercilli*
5	Upper lid raiser	Levator Palpebrae Superioris
6	Cheek raiser	Orbicularis Oculi (pars orbitalis)
7	Lid tightener	Orbicularis Oculi (pars palpebralis)
9	Nose wrinkler	Levator Labii Superioris Alaeque Nasi*
12	Lip corner puller	Zygomatic Major*
15	Lip corner depressor	Depressor Anguli Oris (Triangularis)
16	Lower lip depressor	Depressor Labii Inferioris
17	Chin raiser	Mentalis*
20	Lip stretcher	Risorius
23	Lip tightener	Orbicularis Oris
26	Jaw drop	Masseter, Temporal and Internal Pterygoid relaxed

*represents muscle measured in our study.

Nevertheless, the impact of the misclassified instances described above is not too large, as our facial expression recognition system showed uniform high scores for precision, recall and F1-score (Figure 7C). This indicates a balanced classification performance, with no significant trade-off between precision and recall for any of the expressions. Such consistent results underscore the robustness of the classification model in recognizing and differentiating between the different facial expressions. This conclusion is also supported by the ROC curve and its area (AUC) (Figure 7D), which demonstrate the model’s ability to achieve a high true positive rate with a very low false positive rate, and a strong capability to distinguish between facial expressions, with good potential for practical applications.

### 4.2 SMSM and LRM complement each other to achieve higher quality estimations of facial keypoint displacements

In predicting the displacement of facial points, the SMSM-LRM method showed the most effective performance as measured by $R^{2}$ and NRMSE metrics (Figure 9). On the other hand, in isolation, the SMSM and LRM models showed indistinguishable $R^{2}$ and NRMSE scores, indicating no significant differences between SMSM and LRM in their performance to estimate displacements of facial keypoints. However, a closer examination of the estimations produced by each model in isolation for individual participants revealed notable distinctions (Figure 8; Supplementary Figure S2). Specifically, SMSM showed difficulty in predicting the displacement of facial points with substantial skin connectivity to other facial points. Here, we used separate local SMSM models for each considered facial point. However, skin and muscle forces in one point may interact with other nearby points, which the SMSM model does not account for. This is evident in the case of the disgust expression across all participants, where SMSM consistently deviated from the measured results in predicting the movement of the outer eyebrow (Figure 8), as this point is relatively close to the inner eyebrow. In contrast, LRM was more successful in estimating displacements in these cases, as it establishes a multivariate relation between sEMG signals and facial point displacements, albeit without considering muscle characteristics. However, LRM predictions exhibited greater fluctuations than those from SMSM and SMSM-LRM. For instance, the LRM estimations of displacement of the inner and outer eyebrow were highly variable for expressions producing large displacements (Figure 8). This may be because large displacements are usually associated with larger sEMG signals, which contain considerable signal-dependent noise (Harris and Wolpert, 1998). Therefore, combining the SMSM and LRM creates a connectivity map between facial points (obtained through multivariate linear regression) informed by muscle and skin mechanics (obtained through SMSM), improving over each method applied in isolation. This approach is supported by results in the field of computer graphics, where models of skin elasticity and connectivity are crucial in achieving realistic and natural facial expressions (Kim et al., 2020; Sifakis et al., 2005; Zhang et al., 2001; Lee et al., 1995; Kähler et al., 2001).

### 4.3 Identified muscle synergies may provide insights for generation of facial expressions

In its current form, the SMSM-LRM model is not directly applicable to the facial expression generation task because it is a forward model of the physics of facial motion. That is, it relates muscle activations (sEMG signals) to the displacement of facial keypoints. However, in the facial generation task, an inverse model of the facial motion is needed: a mapping from desired displacements of facial points (or desired facial expressions) to muscle activations. Here, we notice that the results of the muscle synergy analysis could be used in conjunction with the SMSM-LRM model to build an actual facial expression generation system.

As mentioned above, the different facial expressions are associated with clusters of specific combinations of muscle synergies (Figure 6C). Therefore, it is possible to generate trajectories in the synergy activation space from one expression cluster to another. These trajectories in synergy space can be mapped directly to muscle activations U by using Equation 1 and ignoring the residual term E . These muscle activations could in turn be used as an input to the SMSM-LRM model to produce trajectories for the defined facial keypoints. Therefore, the clusters associated to the different facial expressions in the muscle synergy activation space could act as the inverse model needed by the SMSM-LRM to achieve a desired expression. Moreover, these trajectories in synergy activation space could be used to create transitions between different expressions and between different intensities of a given expression, creating a continuum in the space of facial expressions, as opposed to a discrete encoding of predefined expressions.

Evidently, this approach is not exclusively achievable using the extracted muscle synergies, as transitions between facial expressions could also be defined in a space where the activation of each individual muscle constitutes a different dimension. However, using muscle synergies simplifies the visualization of these transitions, and ensures that the transitions follow realistic muscle coordination patterns, resulting in potentially more natural transitions.

### 4.4 Limitations

Here, we have described an effective system to recognize facial expressions and estimate displacements of facial points based on sEMG measurements, muscle synergy analysis, and a skin-musculoskeletal model enhanced with linear regression. However, there remain some limitations in implementation, experimental and application aspects that should be addressed in future work. In the implementation aspect, the accuracy of facial point displacement measurements needs to be quantitatively verified. We measured the displacement of facial points with a 2D camera using object tracking software. Tracking of the position of objects using vision systems is prone to measurement errors due to changes in the pose of the objects. Methods to alleviate this problem have been addressed in the tracking of facial points by using linear transformations to align the measured points given the coordinates of fixed landmarks (Wu et al., 2021). However, in our experimental setup, electrodes for the measurement of sEMG occluded large portions of the face, making it difficult to stably detect facial landmarks using established algorithms. For this reason, we trained a custom algorithm using DeepLabCut to track facial points that we physically marked using stickers on participants’ faces. We manually labeled a subset of the captured images based on stickers, and used this labeled set to train the DeepLabCut algorithm to track the facial points in unlabeled images. This makes it difficult to evaluate the error in our displacement measurements, as we do not have a ground truth for the predictions obtained by DeepLabCut. However, visual inspection of the tracked facial points in the unlabeled images suggests that the tracking performance is adequate.

An additional limitation regarding implementation aspects is that we only measured sEMG from muscles without measuring their contralateral counterparts. This prevents analyzing more complex facial expressions that may be asymmetric, and may reveal more complex patterns of coordination across muscles. The main obstacle for this problem is the number of electrodes that can be placed on the face without obstructing the placement of other electrodes. In our case, the upper limit in the number of electrodes was close to 7. Therefore, analysis of other types of expressions would require removing electrodes from muscles that may provide valuable information. This may be alleviated by future miniaturization of hardware, or use of intramuscular EMG, but this has obvious disadvantages for participant comfort during the execution of facial expressions.

In the experimental aspect, the inter-subject variability in sEMG measurements revealed some limitations in the muscle synergy analysis. The similarity of muscle synergies across all participants suggests a mostly consistent pattern of muscle activation during the six facial expressions (Figure 6B), which correlates with our experimental design based on FACS. However, the structure of synergy 3 was somewhat variable across all participants. These results may be attributable to at least 3 different factors: individual differences in facial structure across participants, differences in the execution of the task across participants, and inconsistency across participants in the measured sEMG caused by cross-talk (Ekman and Rosenberg, 1997). Furthermore, participants provided feedback that some of the facial expressions were similar and challenging to differentiate, making it easy to confuse them during a single trial. They noted that certain expressions were difficult to perform and were not commonly used in their regular emotional expressions, which could result in different activation of specific muscles. This observation aligns with the findings that the facial expressions used to convey emotions in non-photographic scenarios differ from the classic expressions outlined in the FACS (Sato et al., 2019). These variations in muscle activation and expression habits could be contributing factors to the observed differences in muscle synergies among participants. However, these differences do not seem to severely affect performance in the FER task.

Furthermore, we acknowledge a limitation regarding the reliance on a single operator for muscle palpation, electrode placement, and keypoints identification, without external validation. While the operator underwent extensive training, operator-dependent bias cannot be completely eliminated. Future work should incorporate cross-operator validation or automated placement systems to further enhance the reproducibility and consistency of the experimental protocol.

In the context of muscle fatigue, although participants were given rest periods during each trial and experiment to minimize the risk of fatigue, no quantitative measures were employed to monitor or evaluate the occurrence of muscle fatigue. Despite the potential influence of muscle fatigue, our results demonstrate high performance in both recognition and estimation tasks. Future work should incorporate methods to quantitatively assess muscle fatigue, particularly to explore its impact on outcomes in longer experiments and real-world application scenarios.

In the application aspect, the proposed SMSM-LRM model focused on five facial keypoints, which is significantly fewer than the number typically used in facial landmarks detection tasks. However, the proposed model is able to handle the estimation of additional facial keypoint displacements by combining the musculoskeletal model estimations with the linear regression estimations, which allows us to bypass the physical modeling of point-to-point skin interactions. For instance, while the inner and outer points of the eyebrow are influenced by common muscles, their direct interactions are limited. By incorporating the LRM, we can effectively model these types of interrelationships, thereby improving the accuracy of predictions for additional keypoints. We recognize the benefits of including more keypoints and are exploring advances that may allow us to expand our model in future studies. Additionally, we plan to augment the dynamics of the proposed model by employing dynamic models (Chen et al., 2024; Xu et al., 2024), focusing on the transition duration between different facial expressions in our future research.

Finally, regarding the application aspect, the system we propose here is not currently a feasible alternative to vision-based FER. As mentioned above, the placement of the sEMG electrodes makes it difficult to integrate our proposed system into practical applications, especially those involving a direct interaction with a robotic agent. Other studies have explored attaching sEMG sensors to virtual reality (VR) headsets, which could be useful for facial expression generation in VR environments to bypass the use of real-time camera capture, but the placement of the electrodes is limited to the area of the face that the headset covers (Wu et al., 2021). Therefore, new headset designs and further work into the miniaturization of sEMG electrodes could increase the applicability of our system in VR.

## 5 Conclusion

This study presents a framework for facial expression recognition and generation using facial sEMG signals in the realm of Human-Robot Interaction (HRI). We used muscle synergy analysis to accurately recognize facial expressions and developed a skin-musculoskeletal model with linear regression (SMSM-LRM) to predict the displacement of facial keypoints. We achieved significant advancement in performance in a facial expression recognition task based on sEMG signals and muscle synergy activations. The extracted muscle synergies offer a more detailed understanding of the coordination of muscles during the execution of facial expressions. Additionally, our proposed SMSM-LRM shows high fidelity in estimating facial point displacements, showing potential as a useful tool in the field of facial expression generation. Specifically, the relation between muscle activity and facial motions extracted by our model could create the basis to study relationships between muscle synergies and the coordinated motion of facial keypoints. This could be applied to develop a library of controllers for facial actuators in expressive robots that produce more human-like facial motions. By combining muscle synergy analysis and skin-musculoskeletal dynamics, we provide a new perspective in understanding and replicating human facial expressions, paving the way for more expressive humanoid robots, potentially enhancing human-robot interactions.

## Data Availability

The data presented in this study are available upon reasonable request from the corresponding author.
