# Supplementary material for: Facial expression recognition through muscle synergies and estimation of facial keypoint displacements through a skin-musculoskeletal model using facial sEMG signals
Source: Front Bioeng Biotechnol. 2025 Feb 12;13:1490919. doi: 10.3389/fbioe.2025.1490919 (PMC11861201; doi:10.3389/fbioe.2025.1490919)
Supplement: Supplementary file 1 [file DataSheet1.pdf]

Supplementary Material

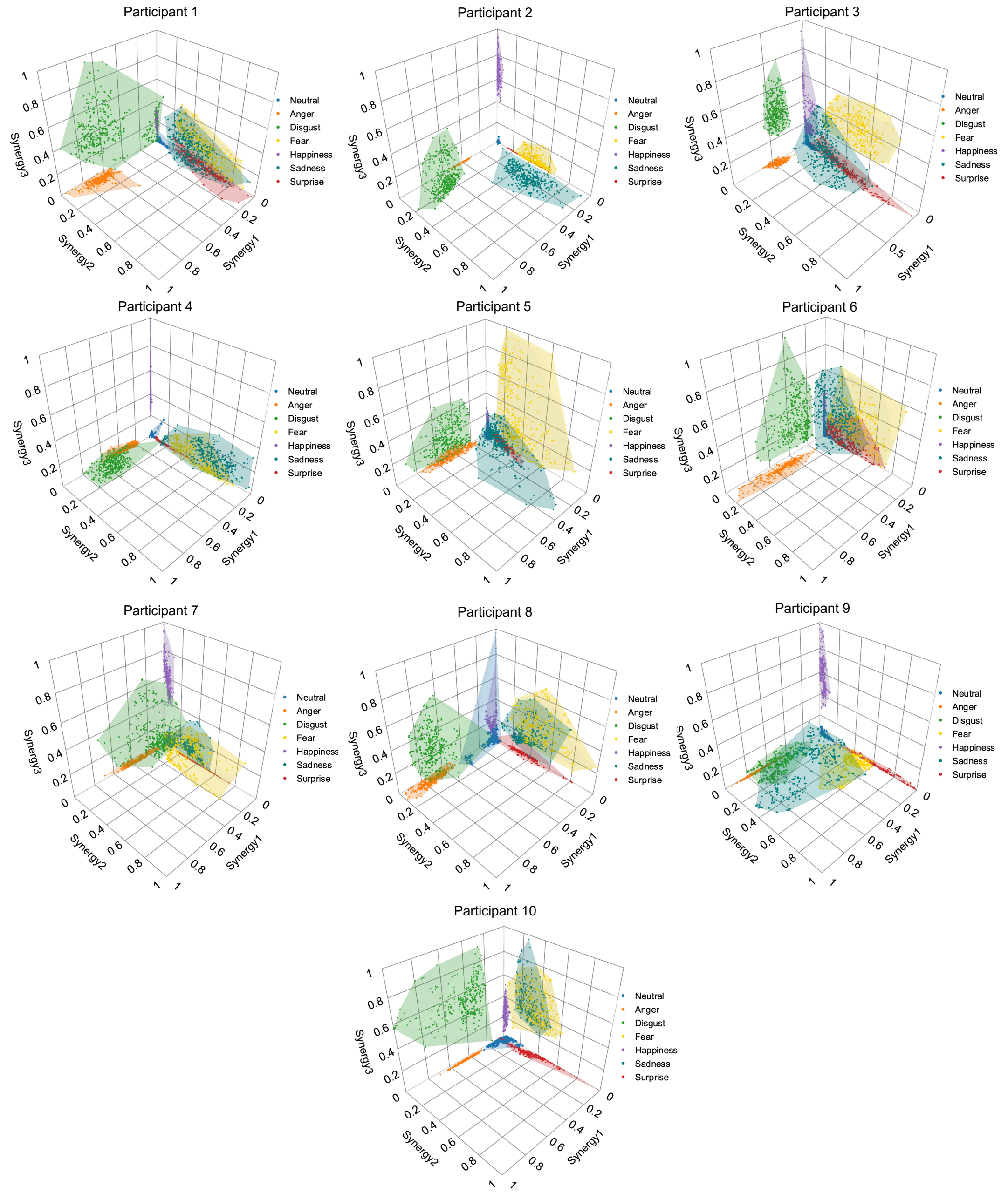

Figure S1. Cluster of activation coefficients in 3 synergy coordinates: Participant 1-10. The shaded areas in the figure represent the convex hulls corresponding to muscle synergy activations for each facial expression. These hulls are computed in three-dimensional space from the points representing the activations and reflect the range and shape of each expression within the muscle synergy activation space. The convex hulls are calculated using the ConvexHull function from the *scipy* library, based on the activation data points for each facial expression, providing a visual representation of the outer boundaries of the muscle activation patterns for each expression.

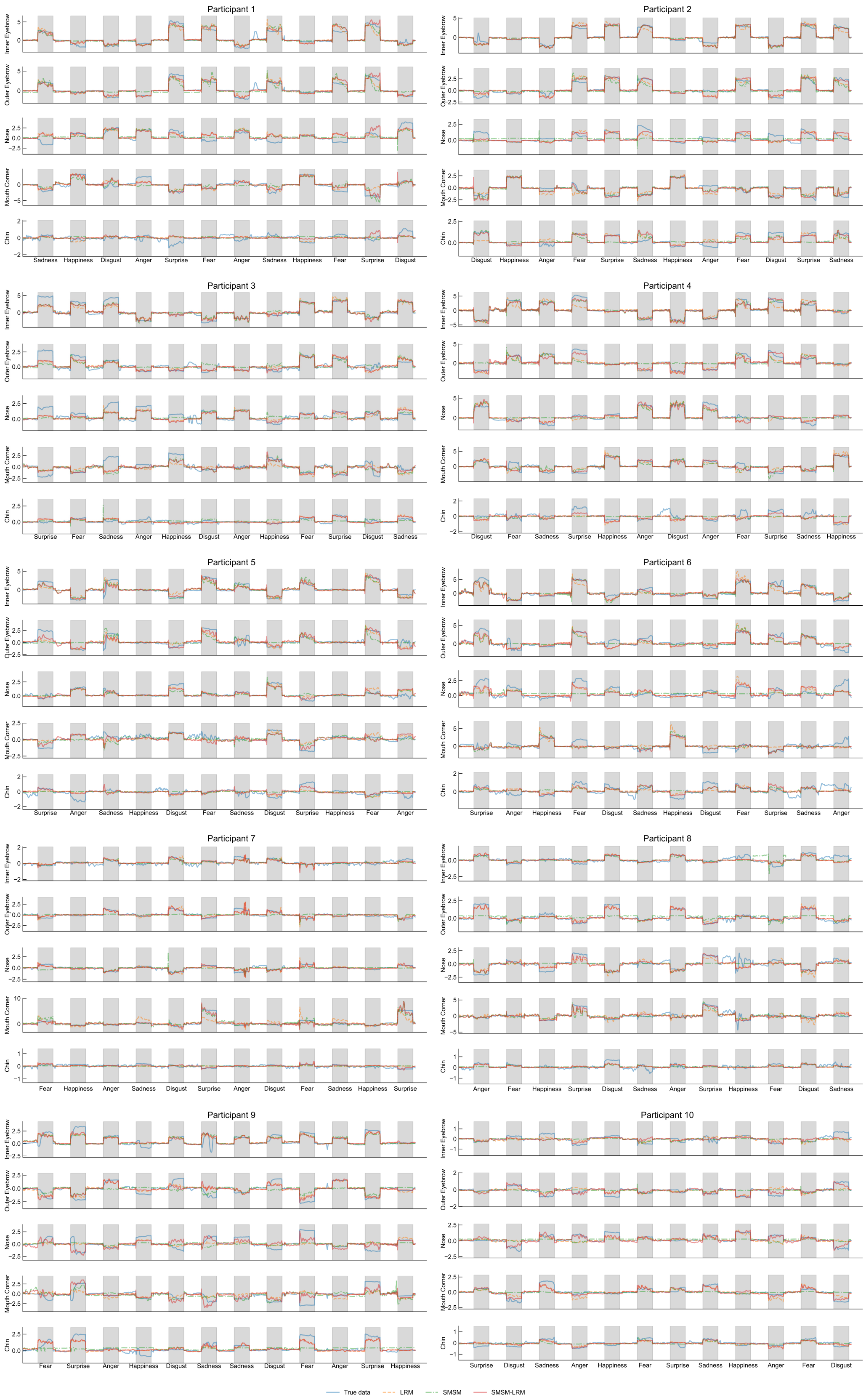

Figure S2. Estimation results of facial keypoints by SMSM, LRM, and SMSM-LRM : Participant 1-10. The displacement of the inner eyebrow, the outer eyebrow, the nose, the mouth corner, and the chin, respectively. The blue lines represented the measured displacements of five facial points calculated based on DeepLabCut. The orange dashed, green dotted, and red lines represent the prediction results from the LRM, SMSM, and SMSM-LRM, respectively.
